# A global dataset of demosponge distribution records

**DOI:** 10.1016/j.dib.2024.110200

**Published:** 2024-02-20

**Authors:** Ariadni Vafeiadou, Eliza Fragkopoulou, Jorge Assis

**Affiliations:** a Centre of Marine Sciences (CCMAR-CIMAR), University of the Algarve, 8005-139 Faro, Portugal; b Faculty of Bioscience and Aquaculture, Nord Universitet, Postboks 1490, Bodø, Norway

**Keywords:** Marine biodiversity, Foundational biodiversity information, Global biogeography, Demospongiae occurrence records, Sponges

## Abstract

Biodiversity information in the form of species occurrence records is key for monitoring and predicting current and future biodiversity patterns, as well as for guiding conservation and management strategies. However, the reliability and accuracy of this information are frequently undermined by taxonomic and spatial errors. Additionally, biodiversity information facilities often share data in diverse incompatible formats, precluding seamless integration and interoperability. We provide a comprehensive quality-controlled dataset of occurrence records of the Class Demospongiae, which comprises 81% of the entire Porifera phylum. Demosponges are ecologically significant as they structure rich habitats and play a key role in nutrient cycling within marine benthic communities. The dataset aggregates occurrence records from multiple sources, employs dereplication and taxonomic curation techniques, and is flagged for potentially incorrect records based on expert knowledge regarding each species’ bathymetric and geographic distributions. It yields 417,626 records of 1,816 accepted demosponge species (of which 321,660 records of 1,495 species are flagged as potentially correct), which are provided under the FAIR principle of Findability, Accessibility, Interoperability and Reusability in the Darwin Core Standard. This dataset constitutes the most up-to-date baseline for studying demosponge diversity at the global scale, enabling researchers to examine biodiversity patterns (e.g., species richness and endemicity), and forecast potential distributional shifts under future scenarios of climate change.

Specifications Table

**Table:** 

Subject	Biodiversity
Specific subject area	Marine macroecology, marine biogeography, biodiversity data, marine conservation and management, climate change assessments
Data format	Excel files (Raw, Filtered)
Type of data	Table, Chart, Graph, Figure
Data collection	Georeferenced occurrence records of the class Demospongiae, dereplicated, taxonomically curated, flagged for potentially incorrect entries regarding each species’ bathymetric and geographic distributions based on expert knowledge available in major databases of biological traits, and standardized with Darwin Core Standard. Data were processed using R statistical computing software, version 4.2.2 (2023).
Data source location	Institution: CCMAR- Centre of Marine Sciences City/Town/Region: Faro, Algarve Country: Portugal Occurrence records of demosponge species compiled from the biodiversity information facilities: (1) Ocean Biodiversity Information System (https://obis.org) (2) Global Biodiversity Information Facility (https://www.gbif.org) (3) Deep-Sea Coral & Sponge Map Portal, National Oceanic and Atmospheric Administration (https://www.ncei.noaa.gov/maps/deep-sea-corals/mapSites.htm) (4) National Biodiversity Network, NBN atlas (https://nbnatlas.org/) (5) Vulnerable Marine Ecosystems, International Council for the Exploration of the Sea (https://vme.ices.dk/download.aspx) (6) PANGAEA – Data Publisher for Earth & Environmental Science (https://www.pangaea.de) (7) BioTIME, A database of biodiversity time series for the Anthropocene (https://biotime.st-andrews.ac.uk) (8) Integrated Digitized Biocollections (https://www.idigbio.org) (9) European Marine Observation and Data Network (EMODnet) – Data Ingestion Portal (https://www.emodnet-ingestion.eu/) (10) Aquamaps, a global online database containing standardized distribution maps for marine species (https://aquamaps.org) Expert knowledge of demosponge species compiled from the biodiversity information facility: (10) Aquamaps, a global online database containing standardized distribution maps for marine species (https://aquamaps.org) (11) SeaLifeBase, a global online database of information about marine life (https://www.sealifebase.ca)
Data accessibility	Repository name: Data identification number: 10.6084/m9.figshare.24591012 Direct URL to data: https://doi.org/10.6084/m9.figshare.24591012

## 1 Value of the Data

• The most up-to-date dataset of demosponge distribution records at a global scale. Marine sponges are keystone components of marine benthic communities, promoting biodiversity thought the provisioning of habitat for numerous organisms, and influencing nutrient cycling [1]. Additionally, they constitute a valuable source of natural products with various applications in biomedical research, pharmaceuticals, and biotechnology [2]. Yet, sponges face numerous threats from environmental changes and human activities, including deep-sea industrialization and fishing. Considering their ecological role and sensitivity to human disturbances, sponges are considered indicator species of Vulnerable Marine Ecosystems (VMEs) in the deep sea [3].
• The dataset is curated, ensuring that records are dereplicated and standardized taxonomically. It includes flags for potentially incorrect records and it is made available under the FAIR principle in Darwin Core Standard. This facilitates smooth integration into statistical analyses and promotes interoperability across biodiversity datasets.
• The dataset serves as a foundational reference for describing species distributions at the global scale and exploring niche-related inquiries, which comprise projections of climate-induced range shifts across space and time [4]. It can also be used in modelling applications to identify suitable habitats of overlooked species and assist in locating VME in poorly known regions [3,5].
• The dataset can assist researchers in tackling priority questions associated with demosponges macroecology, biogeography and climate change responses and impacts. It can assist in unveiling biodiversity patterns such as endemicity centers and species richness hotspots [6], which together can support the implementation of well-informed strategies for conserving, managing, and restoring marine biodiversity.

## 2 Background

Macroecology, biogeography and conservation research rely heavily on complete and precise occurrence data describing the distribution of species [7]. Although open-access biodiversity databases like the Ocean Biodiversity Information System [8] provide access to such information, they often contain spatial and taxonomic errors and can be incomplete. Additionally, the presence of duplicated data in various formats hampers seamless integration and interoperability [9]. Here, we provide a dataset of demosponge distribution records at the global scale, comprising dereplicated records of 1816 taxonomically standardized species and incorporating a quality control system flagging potentially incorrect records [10]. Data are made available under the FAIR principle of Findability, Accessibility, Interoperability and Reusability in the Darwin Core Standard [11].

## 3 Data Description

The dataset of occurrence records of species belonging to the class Demospongiae is provided in Excel format. Rows refer to occurrence records and columns are compatible with the data fields of Darwin Core Standard [11], with a focus on the date, source, location of records, taxonomy, and finally quality flag of records (Table 1).

**Table 1:** Data fields of the global dataset of demosponge distribution records (Additional information on Darwin Core Standard [11]: https://dwc.tdwg.org).

Field	Description
aphiaID	Identifier of the taxon, linked to the World Register of Marine Species
scientificName	Name of the taxon, as originally reported
acceptedName	Accepted name of the taxon, retrieved from the World Register of Marine Species
kingdom	Higher taxonomic classification
phylum	Higher taxonomic classification
class	Higher taxonomic classification
order	Higher taxonomic classification
family	Higher taxonomic classification
genus	Higher taxonomic classification
decimalLongitude	Geographical longitude in decimal degrees of the record's location
decimalLatitude	Geographical latitude in decimal degrees of the record's location
coordinateUncertaintyInMeters	Distance (in meters) from the decimal Latitude and decimal Longitude that describes the center of the circle containing the record's location
depthAccuracy	Depth uncertainty of the record (in meters), as originally reported
locality	Name of the record's location
minimumDepthInMeters	Minimum depth of the record (in meters), as originally reported
maximumDepthInMeters	Maximum depth of the record (in meters), as originally reported
year	Four-digit year in which the observation occurred
month	Two-digit month in which the observation occurred
day	Two-digit day in which the observation occurred
bibliographicCitation	Bibliographic reference of the record
license	“A legal document giving official permission to do something with the resource”
georeferenceProtocol	A description or reference to the methods used to determine the spatial footprint, coordinates, and uncertainties.
scientificNameAuthorship	Authorship information for the scientificName
taxonomicStatus	The status of the use of the scientificName as a label for a taxon.
coordinatePrecision	A decimal representation of the precision of the coordinates given in the decimalLatitude and decimalLongitude.
country	The name of the country or major administrative unit in which the Location occured
individualCount	The number of individuals represented present at the time of the Occurrence.
basisOfRecord	The specific nature of the data record.
measurementOrFact	Quality control based on the flagging system: flagGeographicRange ‘-1’ for records outside the known geographic distribution of species flagVerticalRange ‘-1’ for records outside the known depth range of species flagLand ‘-1’ for records over land

**Fig. 1:**
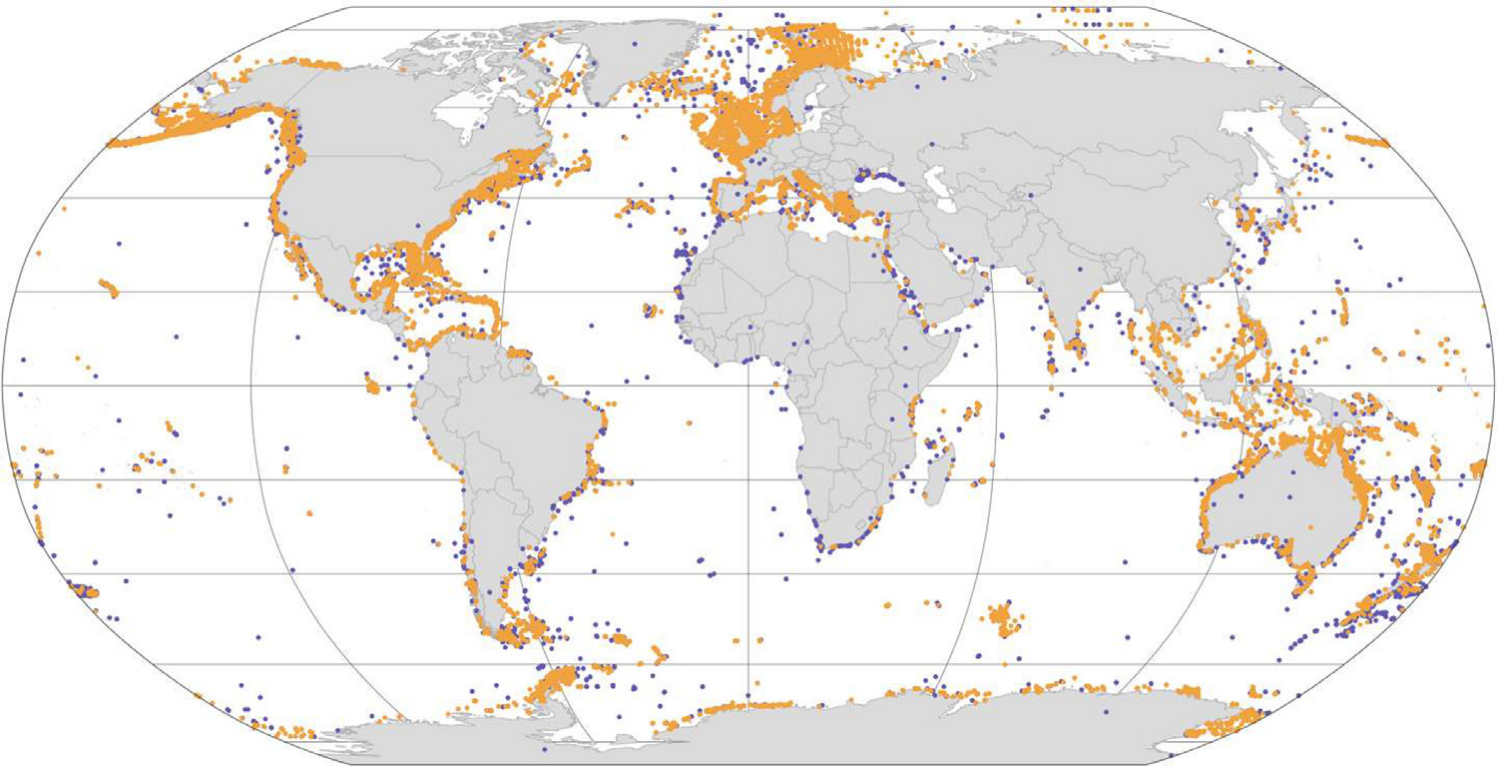
Global map of demosponge records. Points in orange represent occurrences that are flagged as correct, while points in purple indicate potentially inaccurate records based on their known vertical and bathymetric ranges and/or on land.

**Fig. 2:**
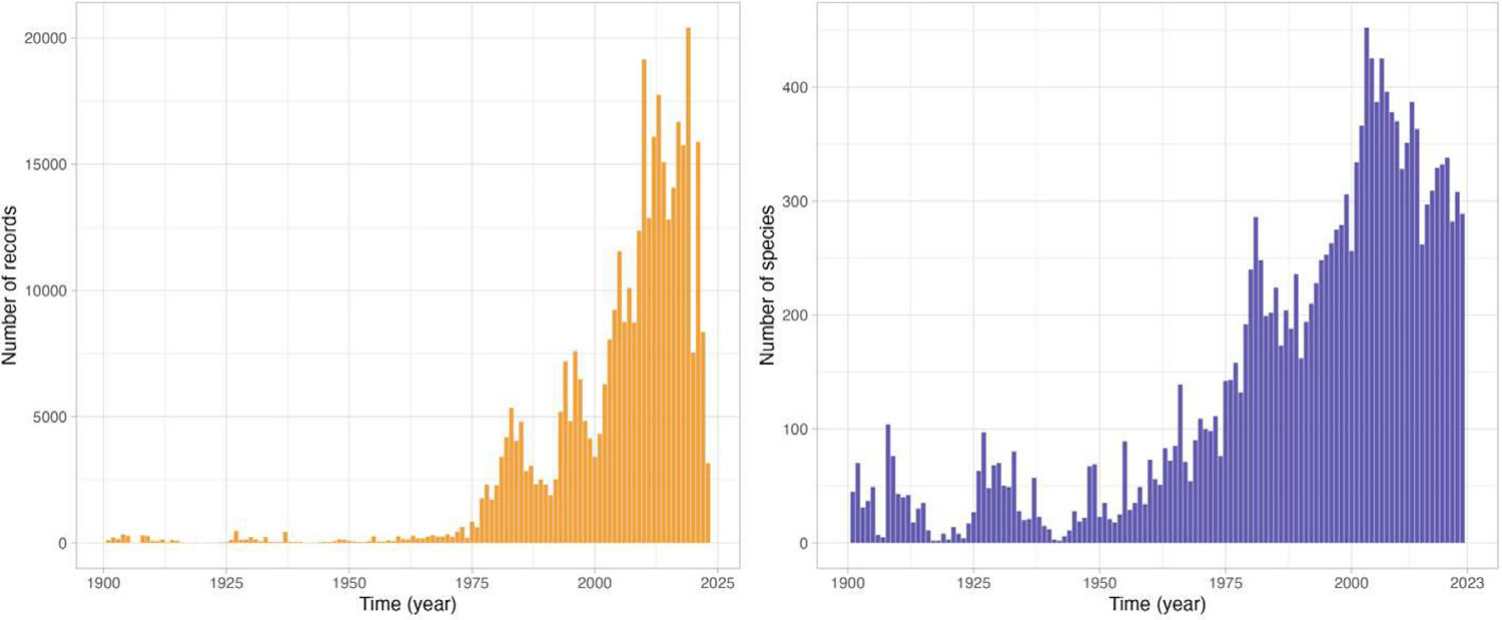
Number of demosponge (a) records and (b) species available in the demosponge dataset per year (data are available since the year 1776. To improve visualization, the few records before 1900 were removed from the graph).

At first, 4776,338 records of occurrence of species belonging to the class Demospongiae were gathered from online biodiversity databases. Records were taxonomically standardized using the World Register of Marine Species, and duplicated and non-georeferenced records were removed. This resulted in a dataset with 417,626 records of 1816 species. Expert knowledge on the bathymetric and geographical distribution of species belonging to the class Demospongiae was gathered from the SeaLifeBase [12], an online database with information about marine life, and Aquamaps [13], a database providing expert-curated species range maps. Only species with current expert knowledge were further considered. Occurrence records falling outside the known bathymetric and geographical distribution, as well as on land, were then flagged as potentially incorrect, resulting in a pruned dataset with 321,660 records of 1495 species belonging to 257 genera, 86 families and 21 orders of the Class Demospongiae (Table 2, Fig. 1), and covering the period from 1776 to 2023 (Fig. 2) and a depth range from 0 to 4820 m [14].

**Table 2:** Number of species, records and flagged records falling (1) over land or out of the known (2) bathymetric and (3) geographical distribution. Numbers in parentheses represent percentages.

Order	Species	Records	Flagged		
			On land	Bathymetric range	Geographical range
Agelasida	26	5368	3 (0.06)	2184 (40.69)	103 (1.92)
Axinellida	116	29,015	108 (0.37)	4047 (13.95)	3338 (11.5)
Biemnida	30	1692	3 (0.18)	314 (18.56)	185 (10.93)
Bubarida	38	4994	21 (0.42)	687 (13.76)	356 (7.13)
Chondrillida	17	5909	38 (0.64)	539 (9.12)	639 (10.81)
Chondrosiida	5	4202	72 (1.71)	45 (1.07)	171 (4.07)
Clionaida	72	46,730	369 (0.79)	2246 (4.81)	2316 (4.96)
Dendroceratida	27	4627	44 (0.95)	511 (11.04)	1025 (22.15)
Desmacellida	9	89	-	19 (21.35)	29 (32.58)
Dictyoceratida	107	41,168	223 (0.54)	4137 (10.05)	4061 (9.86)
Haplosclerida	292	37,001	443 (1.2)	8683 (23.47)	2951 (7.98)
Merliida	9	77	–	16 (20.78)	15 (19.48)
Poecilosclerida	518	69,566	241 (0.35)	7598 (10.92)	8297 (11.93)
Polymastiida	34	19,977	54 (0.27)	2623 (13.13)	1845 (9.24)
Scopalinida	13	2659	4 (0.15)	689 (25.91)	209 (7.86)
Sphaerocladina	1	13	-	7 (53.85)	–
Suberitida	122	73,795	575 (0.78)	5111 (6.93)	8948 (12.13)
Tethyida	46	6694	60 (0.9)	1306 (19.51)	1140 (17.03)
Tetractinellida	303	51,453	89 (0.17)	11,380 (22.12)	12,308 (23.92)
Trachycladida	2	170	2 (1.18)	2 (1.18)	143 (84.12)
Verongiida	29	12,427	173 (1.39)	2575 (20.72)	1768 (14.23)
Total	1816	471,626	2522 (6.03)	54,719 (13.10)	49,847 (11.94)

The global dataset of demosponge distribution records [10] is publicly available in a permanent repository (https://doi.org/10.6084/m9.figshare.24591012) containing 2 main Excel files:

(1) The flagged database, comprising all records.
(2) The pruned database, comprising only records flagged as correct based on each species' known geographic and bathymetric distribution range, and over land.

## 4 Experimental Design, Materials and Methods

The collection and curation steps of the global dataset of demosponge distribution records follow previous studies [9,15] and are detailed below.

Step 1. Collating the list of sponge species belonging to the Class Demospongiae

The taxonomy of sponges covers a broad spectrum of species. The scope of this dataset is focused on marine species of the class Demospongiae, the largest sponge class comprising 81% of all sponges [10]. A list of taxonomically accepted species of the class Demospongiae was collated from the World Register of Marine Species (WoRMS) [16] and was used to search for occurrence records. WoRMS is an authoritative reference system for marine species that offers a unique identification code (aphiaID) associated with a standardized accepted name, and related taxonomic information.

Step 2. Acquisition of occurrence records

Occurrence records of the targeted species were collected from 10 major online biodiversity databases: (1) Ocean Biodiversity Information System [8], (2) Global Biodiversity Information Facility [17], (3) Deep-Sea Coral & Sponge Map Portal, National Oceanic and Atmospheric Administration [18], (4) National Biodiversity Network, NBN atlas [19], (5) Vulnerable Marine Ecosystems, International Council for the Exploration of the Sea [20], (6) PANGAEA – Data Publisher for Earth & Environmental Science [21], (7) BioTIME, A database of biodiversity time series for the Anthropocene [22], (8) Integrated Digitized Biocollections [23], (9) European Marine Observation and Data Network, Data Ingestion Portal [24], (10) Aquamaps [13]. The original source of each record is reported in the respective fields of the Darwin Core Standard.

The dataset exclusively contains occurrence records that are either copyright-free and unrestricted for use or allow any use with appropriate attribution (e.g., CC0 or CC BY, www.creativecommons.org).

Step 3. Taxonomic curation

Taxonomic standardization was performed for each entry with the WoRMS [16]. Entries with status other than accepted were matched with the currently valid species names. Records were also checked to belong to the Demospongiae class, and if not, they were discarded from the dataset.

Step 4. Pruning of occurrence records

Records lacking coordinated information were discarded from the dataset. Additionally, duplicate records of the same species, and sharing the same spatial (longitude, latitude, depth) and temporal information (year, month, day) were discarded from the dataset.

Step 5. Quality control flagging of occurrence records

The large volume of records requires the establishment of a quality control system that can flag potentially incorrect records, which could inadvertently be propagated across repositories via automatic interoperability, despite their source being considered reliable [9]. To address this concern, a quality control protocol, as outlined by Assis et al., 2020 [9,15], was applied to flag records on land and/or with geographical and depth distributions outside currently known species information.

Records over land were identified with a polygon provided by Natural Earth [25], a public domain map that encompasses different scales. Here, the 1:10 m scale layer was employed as a reference. The criterion for flagging records was based on a 1 km Euclidean distance from the ocean, as in Assis et al., 2020 [9].

Additionally, the depth of each record was extracted based on the General Bathymetric Chart of the Oceans, a global terrain model providing elevation data, in meters, on a 15 arc-second interval grid [26]. The depth values were compared to the known bathymetric distribution of the corresponding species based on expert knowledge information provided by SeaLifeBase [12] and Aquamaps [13]. More specifically, records were flagged when their depth values fell out of their known bathymetric range. Likewise, the validation of geographical locations, based on longitude and latitude, was compared to the expert knowledge information for the corresponding species provided by SeaLifeBase [12] and Aquamaps [13]. Known geographical locations were reported in the form of Food and Agriculture Organization (FAO) Major Fishing Areas [27].

Step 6. Dataset format standardization

The dataset was aligned with the Darwin Core Standard, which provides a framework comprising identifiers, labels, and specific definitions to facilitate the exchange of information about biodiversity [11]. The dataset provides standardized information for each record, on source, taxonomy, date, location, depth and quality flag (Table 1).

## Limitations

The dataset may contain the following limitations. Firstly, its taxonomic curation was based on the information available in WoRMS [16]. However, considering that taxonomic statuses may change as new species are continually being discovered and described, WoRMS may not yet contain all recent updates. Secondly, the quality control flagging was based on expert knowledge information provided by SeaLifeBase [12] and Aquamaps [13]. However, these may change as more information becomes available.

## Ethics statement

The present work complies with ethical requirements and does not involve human subjects, animal experiments, or any data collected from social media platforms. No permission was required to use the primary data sources, as they were either copyright-free and unrestricted to use or allowed any use with appropriate attribution.

## CRediT authorship contribution statement

**Ariadni Vafeiadou:** Conceptualization, Data curation, Writing – original draft. **Eliza Fragkopoulou:** Conceptualization, Writing – original draft. **Jorge Assis:** Conceptualization, Data curation, Writing – original draft, Supervision.

## Data Availability

Global demosponge diversity dataset (Original data) (Figshare).
